# Current Understanding of Long-Term Cognitive Impairment After Sepsis

**DOI:** 10.3389/fimmu.2022.855006

**Published:** 2022-05-06

**Authors:** Ying Li, Muhuo Ji, Jianjun Yang

**Affiliations:** ^1^ Department of Anesthesiology, Jiangyin Hospital, Affiliated to Southeast University Medical School, Jiangyin, China; ^2^ Department of Anesthesiology, The Second Affiliated Hospital, Nanjing Medical University, Nanjing, China; ^3^ Department of Anesthesiology, Pain and Perioperative Medicine, The First Affiliated Hospital of Zhengzhou University, Zhengzhou, China

**Keywords:** sepsis, cognitive impairment, BBB dysregulation, neuroinflammation, neurotransmitter dysfunction, neuronal loss

## Abstract

Sepsis is recognized as a life-threatening multi-organ dysfunction resulting from a dysregulated host response to infection. Although the incidence and mortality of sepsis decrease significantly due to timely implementation of anti-infective and support therapies, accumulating evidence suggests that a great proportion of survivors suffer from long-term cognitive impairment after hospital discharge, leading to decreased life quality and substantial caregiving burdens for family members. Several mechanisms have been proposed for long-term cognitive impairment after sepsis, which are not mutually exclusive, including blood-brain barrier disruption, neuroinflammation, neurotransmitter dysfunction, and neuronal loss. Targeting these critical processes might be effective in preventing and treating long-term cognitive impairment. However, future in-depth studies are required to facilitate preventive and/or treatment strategies for long-term cognitive impairment after sepsis.

## Introduction

The central nervous system (CNS) is one of the most vulnerable organs affected by sepsis (1, 2).

Accumulating evidence has suggested that sepsis survivors display long-term neurological sequelae. In particular, sepsis is associated with a 3-fold increase in the prevalence of cognitive impairment (1, 2), which mainly involves declarative memory, working memory, processing speed, and executive function. Generally, the degree of cognitive impairment is affected by poorer pre-sepsis health status, severity of sepsis, and quality of hospital treatment (1). However, in clinical practice, various risk factors also influence the occurrence of long-term cognitive impairment after sepsis. For instance, increased antibiotic treatment latency, delirium, dependence on mechanical ventilation, and long hospitalizations are associated with poor cognitive performance after discharge (3–6). Several animal models mimicking sepsis, including lipopolysaccharide (LPS) injection, cecal slurry injection, or cecal ligation puncture (CLP), have been widely applied in numerous preclinical studies. Nevertheless, the exact pathogenesis of long-term cognitive impairment after sepsis is still poorly understood. Here, we review multiple mechanisms underlying long-term cognitive impairment after sepsis. It is anticipated that future in-depth studies will pave the way for the development of preventive and/or treatment strategies for long-term cognitive impairment after sepsis.

## Blood-Brain Barrier Dysregulation

Recently, the involvement of BBB dysregulation in cognitive impairment after sepsis has been a focus in this field (7, 8). The BBB is a tightly sealed interface between the peripheral circulation and the neuronal cells that is composed of capillary wall endothelial cells, pericytes, and astrocytes. Furthermore, the major components of the BBB and other central nervous system (CNS) cell types form a dynamic structure called the neurovascular unit, which is also critical for normal cognitive function (9). An intact BBB is essential for keeping pathogens, pro-inflammatory signals, and neurotoxic substances outside the brain (10). However, in the sepsis animal model induced by LPS injection, the function of pericytes and endothelial cells is significantly disrupted, leading to BBB damage and cognitive dysfunction (11, 12). Also, in patients suffering from critical illness, markers of BBB injury, such as S100β, are elevated and negatively associated with global cognition at 3 and 12 months after hospital discharge (13). At the molecular level, unlike peripheral endothelium, the BBB endothelium is sealed by tight junctions (TJs), restricting the paracellular routes for diffusion of polar molecules and macromolecules (14). Claudins and occludins are principal TJ proteins bridging adjacent endothelial cells while zonula occludens family (ZO-1, -2, and -3) are scaffolding proteins that provide structural adaptors of TJs (10). *In vitro* studies revealed decreased claudin 5, occludins, and ZO-1 levels in response to LPS stimulation in a concentration-dependent manner (15–18). A recent human autopsy study showed that claudins and occludins were lost in the cerebral endothelium of patients with fetal sepsis (19). Adherens junctions (AJs), another critical aspect of the BBB, connect the actin cytoskeleton of neighboring endothelial cells and thus strengthen the endothelial integrity of the BBB (20). Importantly, AJs modulate the passage of inflammatory cells such as lymphocytes, monocytes, and neutrophils in systemic inflammation (10). AJs are mainly composed of vascular endothelial cadherin and platelet endothelial cell adhesion molecule-1, the dysregulation of which are responsible for BBB hyperpermeability under inflammatory conditions (21, 22). Gap junctions enable exchange of electrical and metabolic signals between endothelial cells and astrocytes (23), which are dynamically changed in response to extracellular stimulations (24).

Many agents are effective in maintaining BBB integrity and have drawn increasing attention for use in preventing long-term cognitive impairment after sepsis. Biologically, hydrogen (H2) is a selective antioxidant that has been tested for the treatment of graft-versus-host disease, aplastic anemia, and hemorrhage (25). Its low molecular weight makes it easy for H2 to diffuse the BBB (26). Yu and colleagues (27) confirmed that immediate inhalation of 2% H2 after CLP decreased escape latency of mice in the Morris water maze (MWM). Intriguingly, H2 had no protective effect when nuclear factor erythroid-2-related factor 2 (Nrf2) was knocked out in the septic mouse model, indicating that Nrf2 is involved in its protective mechanism. The same group demonstrated improvement of spatial memory in the MWM after H2 inhalation in a chronic sepsis model of intraperitoneal injection of human stool suspension (28). The impaired BBB was again restored. Importantly, H2 treatment significantly upregulated Nrf2 expression, which further validated their previous findings. It is worth mentioning that the disrupted CA1 structure was rearranged and the lost pyramidal neurons were replenished in the CA1 area of the hippocampus after H2 treatment. However, their studies covered only a time window of 10 to 14 days after sepsis. Maintenance of BBB integrity and other beneficial effects of H2 in a longer period need to be verified in the future.

## Neuroinflammation

Neuroinflammation is a major component of the etiology of numerous neurological and neurodegenerative diseases (29). During sepsis, following the propagation of peripheral inflammation, the pro-inflammatory signals reach the brain *via* impaired BBB segments, and eventually trigger neuroinflammation, which is primarily characterized by the activation of microglia. In general, activated microglia have two distinct phenotypes, a pro-inflammatory M1 phenotype and an anti-inflammatory M2 phenotype. In both septic animals and patients, the activation of M1 microglia is consistently detected and widely investigated (30–32). It has been shown that inhibition of microglial activation with minocycline is sufficient to prevent long-term memory impairment of mice undergoing CLP procedure (33). In detail, microglia are resident macrophages of the brain parenchyma and are endowed with a host of membrane pattern recognition receptors for recognizing different pathogen-associated molecular patterns (PAMPs) and damage-associated molecular patterns (DAMPs) generated during sepsis (34). It is worth mentioning that one of the most important DAMPs is high‐mobility group box 1 (HMGB1), which is released by innate immune cells beyond LPS stimulation. Significantly, its persistent high serum level is involved in the pathogenesis of long-term memory impairments of sepsis survivors (35). Among various pattern recognition receptors, Toll-like receptors (TLRs) play unique roles in innate immunity in sepsis by specifically recognizing LPS and other PAMPs or DAMPs. Following the recognition of PAMPs and DAMPs by TLRs, multiple intracellular signaling pathways are activated, which lead to the activation of IκB kinase (IKK). IKK is then ubiquitinated and degraded. Afterwards, the nuclear localization sequences on NF-κB protein are exposed, freeing NF-κB dimer to translocate into the nucleus, where it binds to its consensus sequence on the promoter regions or enhancer regions of targeted genes and initiates the pro-inflammatory gene expression (36–38). Importantly, microglia express most TLRs in the brain, among which TLR2 and TLR4 have been regarded as the main ones involved in neuroinflammation (39, 40). It has been demonstrated that anti-TLR2 and anti-TLR4 treatment can prevent LPS-induced NF-κB activation (41). However, more studies are needed to provide mechanistic insight into how anti-TLR could be effective for avoiding long-term cognitive impairment after sepsis. Additionally, when the NF-κB signaling was activated by microRNA-301b, microglial activation in the hippocampus was then induced and excessive TNF-α and IL-Iβ were secreted, which eventually led to the disruption of spatial learning and memory (42). On the contrary, prophylactic inhibition of NF-κB by deleting IKKβ in microglia attenuated its activation and reduced the pro-inflammatory cytokines such as IL1β, IL6, and TNF-α (43). Therefore, NF-κB should be a potential target for modulating microglia activation status in sepsis.

The imbalance of kynurenine pathway (KP) of tryptophan metabolism in the CNS is another possible mechanism underlying microglia-related long-term cognitive impairment after sepsis. In the beginning of the KP, tryptophan in the brain is metabolized to kynurenine by 2,3-dioxygenase (IDO). Then, the KP segregates into two major branches. In the “neuroprotective” branch of KP, kynurenine is converted to kynurenic acid (KA) mainly in astrocytes. KA is an endogenous antagonist of the N-methyl-D-aspartate receptor (NMDAR) and α7 nicotinic acetylcholine receptor (α7nAChR), both of which are highly implicated in cognitive function. While in the “neurotoxic” branch, kynurenine is converted to quinolinic acid (QA) predominantly in activated microglia. QA is an NMDAR agonist and can also inhibit reuptake of glutamate by astrocytes, leading to excitotoxicity and neuroinflammation (44). In the context of sepsis-induced neuroinflammation, pro-inflammatory cytokines robustly upregulate the expression and activity of brain IDO, resulting in more production of kynurenine. Meanwhile, those cytokines also promote the diversion of the KP towards QA production from kynurenine. As a result, QA is excessively generated by microglia, leading to brain injury and cognitive impairment (45). Vitamin B6 acts as an important cofactor of the critical enzymes in the KP. Danielski et al. revealed that immediate administration of Vitamin B6 after CLP improved non-associative memory of mice in the object recognition test 10 days later (46). Thus, maintaining the homeostasis of the KP plays a key role in neuroinflammation modulation and may also be a possible therapeutic strategy.

The activation of microglia not only results in the activation of transcription factor NF-κB and subsequent pro-inflammatory cascades, but also induces over-generation of reactive oxidative species (ROS). The production of ROS in microglia is mostly mediated by NADPH oxidase (NOX) (47). Immunohistochemistry and single-cell RNA-sec study indicates that NOX2 is the most common NOX isotype in human and rodent microglia (48, 49). NOX2 activation is associated with oxidant production in sepsis while acute pharmacological inhibition of NOX2 with low dose apocynin could reduce oxidative stress and prevent spatial memory impairment in the MWM 15 days after sepsis (50). Notably, apocynin can eliminate memory deficits in the inhibitory avoidance test (50). Similarly, our previous study suggested that 10 consecutive days of apocynin treatment after CLP rescued associative memory as measured by freezing time in the fear conditioning test (51). Vitamin C has long been viewed as an antioxidant and has been applied to treat infection, cancer, and even COVID-19 most recently (52–55). In sepsis, high doses of Vitamin C 24 hours after CLP protected septic animals, including reduced cerebral inflammation, oxidative injury, and subsequent improved spatial memory in the MWM (56). Dimethyl fumarate is usually prescribed for relapsing multiple sclerosis and psoriasis. Its effect in cellular antioxidation and detoxification has attracted increasing attention (57). Zarbato and colleagues reported that dimethyl fumarate reduced ROS almost to the control level in a CLP rat model and reversed memory deficits in the object recognition task 10 days after surgery (58). In addition, antioxidant therapy is also helpful in ameliorating cognitive dysfunction in direct cerebral infection caused by microbials (59, 60). In the future, large-scale clinical studies are warranted to verify the potential of antioxidants for preventing long-term cognitive impairment after sepsis.

In addition to microglial activation, astroglial activation is also involved in neuroinflammation after sepsis. Like microglia, the astroglial pathology is also primarily mediated by TLRs. TLRs on astrocyte membranes are stimulated mainly through binding myeloid differentiation factor 88 or Toll/interferon-1 receptor domain-containing adaptor inducing interferon-β, followed by the induction of the NF-κB signaling as well (61). Moreover, S100B, a ligand of Receptor for Advanced Glycation End products (RAGE), is secreted by astrocytes in the CNS during sepsis, which is also recognized as a DAMP. High levels of S100β stimulates iNOS in astrocytes *via* activation of the RAGE/NF-κB pathway (62). Although the long-term cognition function was not assessed, Li and colleagues proved that inhibition of S100β/RAGE/NF-κB pathway reduced neuroinflammation, oxidative stress, and reactive gliosis in the hippocampus in the CLP sepsis model (63).

Activation of microglia and astroglia leads to a storm of pro-inflammatory cytokines including IL1-α, IL-1β, TNF-α, IL-6, IL-12, IL-23 inside the cerebral parenchyma (64, 65). On the other hand, the anti-inflammatory cytokines including IL-4 and IL-10 decrease considerably (31). IL-1β is one of the most relevant cytokines for mediating inflammatory injury in sepsis. After secretion, IL-1β causes synapse damage, contributing to short memory deficits in mice (66–68). Intriguingly, the application of IL-1Ra, a competitive inhibitor of IL-1β, can ameliorate synapse loss in cultured neurons in medium derived from cultured microglia with LPS stimulation (66). Moreover, intracisternal injection of IL-1Ra prevented non-associative memory and aversive memory deficits 10 days after CLP (69). These studies indicate that targeting IL-1β is sufficient to prevent long-term cognitive impairment after sepsis. TNF-α is released as a soluble cytokine primarily by microglia in the brain. It’s another crucial terminal product of the neuroinflammation cascade in sepsis. It has been suggested that systemic administration of TNF-α can induce cognitive impairment (70), while anti-TNF-α treatment yields favorable outcomes in diseases with cognitive decline due to neuroinflammation (71–73). In addition, TNF-α-modulating agents such as stains can prevent long-term spatial learning and memory impairment in the MWM in septic animals about 2 weeks after intraperitoneal injection of cecal material or CLP (74, 75). However, clinical trials using anti-TNF-α agents for preventing long-term cognitive impairment are still sparse.

Several other neuroinflammation-related mechanisms of long-term cognitive impairment after sepsis have been recently proposed. For example, neuroinflammation in sepsis is always accompanied by mitochondrial dysfunction which is associated with reduced ATP synthesis, over-production of ROS, and dysregulated apoptosis (76). Indeed, Manfredini et al. proved that activators of mitochondrial biogenesis such as rosiglitazone significantly improved impaired memory of rats 10 days after CLP. CD40-CD40 ligand pathway plays an important role in the neuroinflammation and oxidative stress in a series of disorders. Michels and colleagues concluded that long-term memory deficits identified by the inhibitory test and open-field task can be ameliorated by anti-CD40 treatment (77). Moreover, it has been demonstrated that fish oil-rich emulsion (78) and alpha-lipoic acid (79) may prevent long-term cognitive impairment by attenuating acute neuroinflammation in sepsis. Cerebral monocyte recruitment is another hallmark of neuroinflammation. Mouse monocytes can be divided into two distinct subsets: inflammatory monocytes with Ly6C^high^CCR2^high^CX3CR1^low^ expression and anti-inflammatory monocytes with Ly6C^low^CCR2^low^CX3CR1^high^ (80). Inflammatory monocytes use chemokine receptor CCR2 for their adhesion to vesicular wall and infiltration into the brain (81). In a model of *S. pneumoniae* pneumonia-induced sepsis, intravital imaging of CCR2 reporter mice brain showed an increase in the rolling and adhesion of CCR2^+^ monocytes in the vasculature. This increasing recruitment of CCR2^+^ monocytes into the brain was followed by microglial activation and neuroinflammation 24 hours after infection. Meanwhile, spatial memory deficits, represented by less travel distance and less duration in the target quadrant in the MWM, appeared 2 weeks later and persisted for at least 9 weeks. Remarkably, anti-CCR2 treatment in the acute phase could abolish the rolling and adhesion of CCR2^+^ monocytes and thus prevent the long-term spatial memory deficits in the MWM (82). Taken together, intervention by inhibiting inflammatory reaction especially in the early phase should be considered in clinical practice and may serve as a possible therapeutic strategy.

## Neurotransmitter Dysfunction

**Figure 1:**
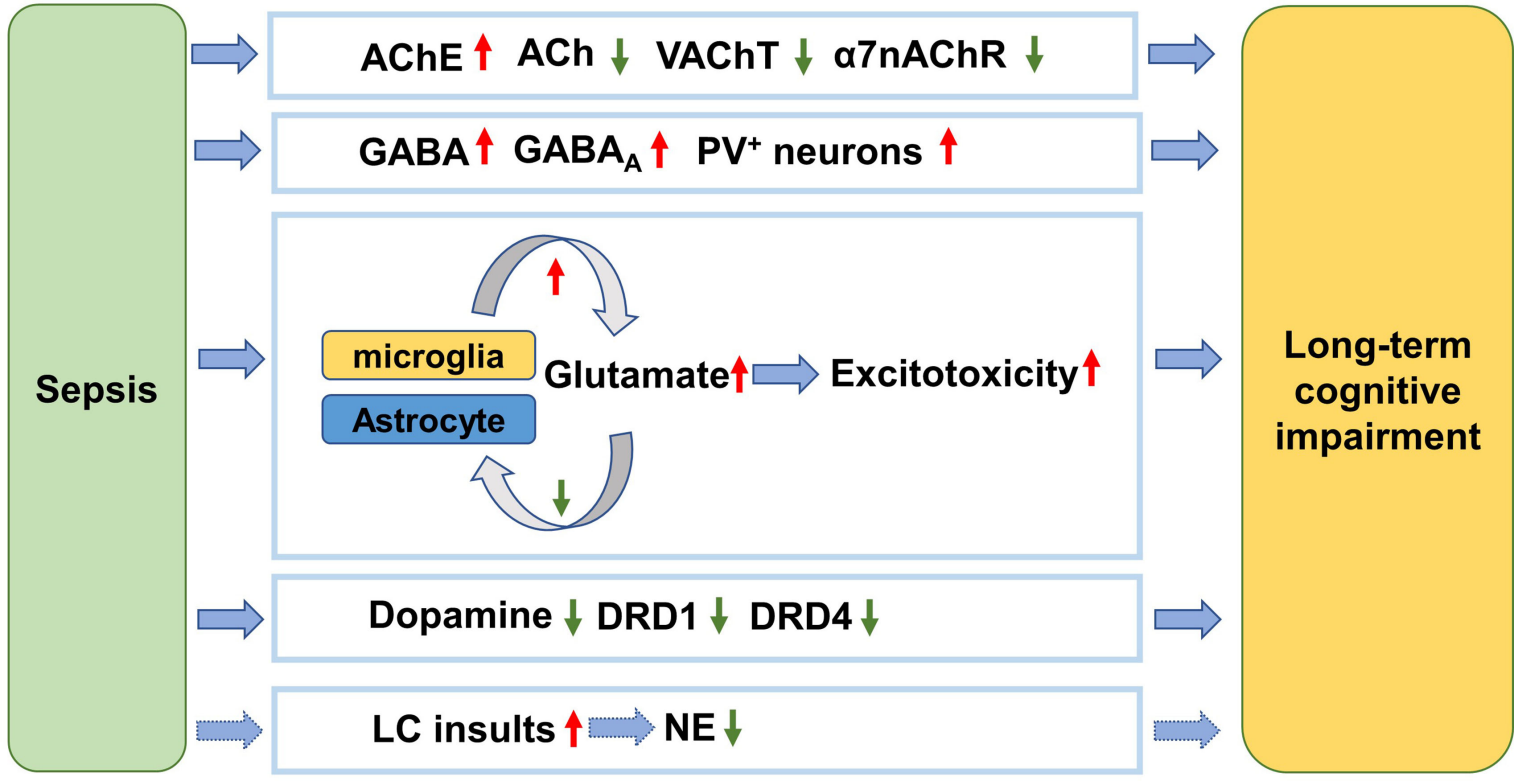
Neurotransmitter dysfunction mechanism underlying long-term cognitive impairment after sepsis. After sepsis, the AChE is activated and the ACh level is thus decreased. Meanwhile the cholinergic innervation is inhibited, as reflected by reduction of VAChT. The α7nAChRs are also downregulated. The suppression of ACh function is associated with cognitive impairment following sepsis. PV^+^ interneurons are activated after sepsis resulting in cognitive impairment. The activated microglia secret more glutamate while the damaged astrocytes reuptake less glutamate, causing excitotoxicity to the brain and cognitive impairment. The level of dopamine is decreased and the receptors are inhibited by sepsis, which is involved in cognitive impairment. The LC is the only source of NE in the brain. NE level decreases in response to sepsis, which contributes to cognitive impairment. AChE, acetylcholinesterase; ACh, acetylcholine; VAChT, vesicular acetylcholine transporter; α7nAChR, α7-nicotinic acetylcholine receptor; GABA, Gamma-aminobutyric acid; GABA_A_, GABA A receptor; PV, parvalbumin; DRD1, dopamine receptor D1; DRD4, dopamine receptor D4; LC, locus coeruleus; NE, norepinephrine.

Neurotransmitter dysfunction plays crucial roles in the pathophysiology of sepsis-associated cognitive decline (**Figure 1**). In the CNS, acetylcholine (ACh) modulates the state of a group of neurons in response to environmental stimulations. Specifically, cholinergic inputs from the medial septum innervate hippocampal circuits and thus regulate hippocampus-dependent cognitive function (83). In fact, acetylcholinesterase (AChE) inhibitors can ameliorate some cognitive symptoms of Alzheimer’s disease by blocking the breakdown of ACh in the brain (84). A significant increase of AChE activity was observed in septic patients experiencing cognitive dysfunction, suggesting that ACh is involved in sepsis-related cognitive impairment (85). In an animal study, the mice made more wrong choices in the 8-arm radial maze test after total recovery from sepsis induced by LPS, which was accompanied by substantial loss of cholinergic boutons in the parietal association and somatosensory cortical areas (86). α7nAChR is the most abundant nicotinic receptor of ACh expressed in the pyramidal neurons and GABAergic neurons in the hippocampus. In particular, α7nAChR plays a distinct role in modulating synaptic activity and plasticity due to its relatively high permeability to calcium. Therefore, α7nAChR agonist has been pursued as a potential therapeutic target for various diseases with cognitive impairments. Recently, selective agonism of α7nAChR has been demonstrated to reduce HMGB1 release and internalization, inhibit the NF-κB pathway (87, 88), attenuate neuroinflammation and significantly increase freezing time of mice in the contextual fear conditioning test (89). Conversely, another study suggests that inhibition of α7nAChR is sufficient to block the protective effects of dexmedetomidine in rescuing impaired cognition in endotoxemia (90). Recently, Tchessalova and Tronson developed a sub-chronic immune challenge model in which they performed intraperitoneal injection of LPS or Polyinosinic:polycytidylic acid intermittently. Using this model, they showed that female mice had significant impairments only in the object recognition, while male mice exhibited broader cognitive impairments in tests including the object recognition, and both context and tone fear conditioning several months later (91). With regard to sex difference, it has been shown that the dysregulated neuronal and synaptic genes in the hippocampus of old male mice recover slower from sepsis than old female mice in the CLP model (92). Interestingly, α7nAChR in the hippocampus of male mice was more vulnerable to sepsis than that of female mice, contributing to worse cognitive performance (93). In addition, genetic knockout of α7nAChRs only worsened the male’s performance in the spatial discrimination task (94). Taken together, α7nAChRs are implicated in the sex difference in susceptibility to long-term cognitive impairment after sepsis. Thus, these findings suggest that sex difference should be taken into consideration when designing α7nAChR-targeting medications for treating sepsis patients.

In the hippocampus, GABAergic interneurons expressing parvalbumin regulate memory and learning (95, 96). Under LPS challenge, the number of parvalbumin-positive inhibitory synapses and frequency of miniature inhibitory postsynaptic currents obviously increased, indicating elevated GABAergic transmission of hippocampal neuronal circuits (97). Concomitantly, the declining cognitive function was identified by spontaneous alternation in the Y maze test, while selective blocking of GABA_A_ receptor could reverse LPS-induced cognitive impairment (97). In contrast to GABA, glutamate is a prevalent excitatory neurotransmitter. In the septic brain, the activated microglia acquire an M1 phenotype and then produces excessive amounts of glutamate into the extracellular space, leading to exacerbated excitotoxicity to neuronal circuits and cognitive deficits. On the other hand, astrocytes are the primary glutamate buffering cells, maintaining the homeostasis of glutamate (98). However, astrocytes are damaged and lost during sepsis, presumably resulting in decreased glutamate uptake (99). Furthermore, astrocytes themselves also secrete glutamate and increase the excitability of adjacent neurons (100). Guanosine exerts neuroprotective effects probably by decreasing extracellular glutamate level and augment astrocytic glutamate uptake. Interestingly, guanosine was proven to eliminate the detrimental effects of CLP on rats’ memory function (101). These results suggest that glutamate dysregulation is involved in cognitive impairment following sepsis.

A recent study reported decreased dopamine levels in the hippocampus after CLP, which was associated with lower discrimination index in the object recognition test 12 days later and increasing escape latency ain the Barnes maze test 14 days later (102). However, intraperitoneal administration of L-dopa/Benserazide hydrochloride (L-DA) overtly reduced the percentage of activated microglia in hippocampus. Meanwhile, mRNA levels of pro-inflammatory cytokines such as TNF-α, IL-1α and IL-1β were significantly downregulated. Meaningfully, L-DA restored memory and learning function of septic mice in the object recognition test and the Barnes maze test. The authors also found that the L-DA’s inhibition of microglial activation was abolished when dopamine D1 receptor antagonist was added. However, it should be noted that L-DA was effective only when it was applied in the acute phase of sepsis (102). Moreover, significant dopamine D4 receptor loss was also reported in mice suffering from cognitive impairment in a CLP model while agonism of D4 was sufficient to rescue cognitive abnormalities (103).

Norepinephrine (NE) is another important neurotransmitter regulating cognition such as episodic memory and working memory (104). To date, there is sparse evidence that directly links NE dysregulation to long-term cognitive impairment after sepsis. In the CNS, most of the NE is released from the locus coeruleus (LC) neurons in the brain stem (105). LC neurons are vulnerable to toxins and inflammation due to their high energetic requirement (106). It can be assumed that overwhelming sepsis would injure LC neurons and thus cause NE deficits. Therefore, it’s worth testing NE modulators such as some antidepressants for preventing long-term cognitive impairment after sepsis in future (106).

## Neuronal Loss

Neuronal loss has been identified to be associated with long-term cognitive impairment in both septic animals and patients (**Table 1**). As for adult animals, Semmler et al. (86) showed that rats supposed to recover from sepsis still had neuronal loss in the CA1 and CA2 of the hippocampus and the prefrontal cortex 12 weeks after LPS exposure. Meanwhile, the rats spent more time in the corners in the open field test and had less correct entries during training trials in the 8-arm radial maze test, indicating impaired cognitive function. In adult mice undergoing CLP, several studies reported progressive and irreversible neuronal loss in CA1 and CA3, which was linked to longer escape latency in the MWM (74, 107, 108). In neonatal rats treated with LPS, loss of dopaminergic neurons in mesencephalic substantia nigra was revealed when they showed long-term impairment in locomotor function and spatial memory (110). In neonatal mice, LPS-induced systemic inflammation resulted in significant cerebellar atrophy as well as neuronal loss in the CA3 and the pons at least for 9 days (99). Interestingly, it was also found that sepsis caused loss of neural stem cell in the DG of mice, which contributed to hippocampal neurogenesis and cognitive impairment (114). Besides direct neuronal loss, Semmler et al. (86) noted that LPS caused a reduction of cholinergic innervation in the parietal cortex. In addition, Huerta et al. (115) provided data showing that CLP induced substantial decline of dendritic spine density of excitatory neurons in the basolateral nucleus of the amygdala and granule cells in the dentate gyrus (DG). Consequently, the mice still had contextual fear memory impairment 78 to 176 days after CLP. Hence, it can be inferred that the loss of neuronal synapse due to sepsis may also be implicated in sustained cognitive impairment. Nevertheless, there are also inconsistent results. For instance, some other researchers argued that mice with long-term cognitive impairment had no significant hippocampal volume reduction in MRI or obvious changes of cellularity of the DG region (82). In the future, novel approaches for detection of neuronal loss locally and globally will broaden our understanding of its role in sepsis-induced long-term cognitive impairment.

**Table 1:** Representative studies of neuronal loss mechanism underlying long-term cognitive impairment after sepsis.

Reference	Subjects	Model/disease	Method for identifying Neuronal loss or brain atrophy	Brain areas with neuronal loss or atrophy	Subtype of lost neurons	Cognitive impairment	Time of evaluation
Semmler et al., (86)	Adult rats	10 mg/kg of LPS intraperitoneally	Neuronal cell count by NeuN staining	CA1, CA2, and the prefrontal cortex.	N/A	Less center occupancy in the open field test; less correct entries in the 8-arm radial maze test.	12 weeks after LPS injection
Liu et al., (107)	Adult mice	CLP	Nissl staining	CA1, CA3,	N/A	longer escape latency, less time and crossings in the target quadrant in the MWM.	2 weeks after CLP
Guo et al., (108)	Adult mice	CLP	Nissl staining	CA1	N/A	Longer escape latency in MWM	60 days after CLP
Tian et al., (109)	Adult mice	CLP	Neuronal cell count by HE staining	CA3	N/A	Less total distance, rearing, center occupancy, grooming in the open field test; longer escape latency, less time and crossings in the target quadrant in the MWM.	15-17 days after CLP for the open field test; 19-22 days after CLP for the MWM
He et al., (110)	Neonatal rats	1 mg/kg of LPS intraperitoneally	Neuronal cell count by TH staining	Mesencephalic substantia nigra	TH^+^ dopaminergic neurons	Less total distance and rearing in the open field test; longer escape latency and less time in the target quadrant in MWM.	80-85 days after LPS injection
Gunther et al., (111)	Patients	ICU survivors	MRI	VBR, thalamus, cerebellum, superior frontal lobe,	N/A	Worse global cognitive performance in the global RBANS score, worse executive functioning in the TMT-B and worse visual attention in TMT-A.	12 months after ICU discharge
Semmler et al., (112)	Patients	25 septic and 19 non-septic ICU survivors	MRI	left hippocampus and total hippocampus	N/A	Worse performance in the digit span, 2-back-test, alertness, GoNogo, verbal memory, phon. VF and TMT-B of the Neuro Cognitive Effects; worse learning and more memory loss in the auditory verbal learning test; worse performance in the copy of the Rey complex figure test.	6-24 months after ICU discharge
Seidel et al., (113)	Patients	severe sepsis	BrainAGE score	N/A	N/A	Lower cognitive sum score of the five cognitive domains (alertness, divided attention, selective attention, working memory and verbal memory)	longer than 2 years after sepsis

LPS, lipopolysaccharide; CA1, cornu ammonis 1; CA2, cornu ammonis 2; VAChT, vesicular acetylcholine transporter; N/A, not available; CLP, cecal ligation puncture; MWM, Morris water maze; TH, tyrosine hydroxylase; ICU, intensive care unit; VBR, ventricle-to-brain ratio; TMT-B, Trail Making Test Part B; TMT-A, Trail Making Test Part A; MRI, magnetic resonance imaging; BrainAGE, brain age gap estimation.

As for septic patients, magnetic resonance imaging (MRI) technique has been the first choice for detecting brain atrophy due to neuronal loss. For example, a clinical study investigated the brain volume and cognition of sepsis patients. The results suggested that greater brain atrophy 3 months after sepsis could predict worse cognitive performance at 12 months (111). The affected areas included the frontal lobes, thalamus, and cerebellum. Another MRI study presented evidence of prominent atrophy of the left hippocampus in sepsis survivors who had permanent cognitive deficits in verbal learning and memory 6 to 24 months after discharge from the ICU (112). In patients with sepsis-induced brain dysfunction, Orhun and colleagues (116) found prominent atrophy of gay matter in several limbic structures including the temporal lobe and insula *via* a voxel-based morphometry analysis base on MRI. However, they (116) later identified atrophy of cerebral and cerebellar white matter, cerebral cortex, hippocampus, and amygdala whereas the brainstem, cerebellar cortex, and deep gray matter structures were relatively resistant to septic damage (117). The discrepancy of affected regions of the two studies might be attributed to the sepsis stages of patients, which indicated that the brain may undergo a dynamic alteration in response to sepsis. Based on structural MRI, the “brain age gap estimation” (BrainAGE) score reflects the age-specific grey matter atrophy of the whole brain (118). Seidel et al. (113) followed up 20 patients with severe sepsis and reported that patients suffered from cognitive impairment 2 years later or longer. It is worth mentioning that the severity of the patients’ cognitive impairment was closely associated with their BrainAGE scores (113).

**Figure 2:**
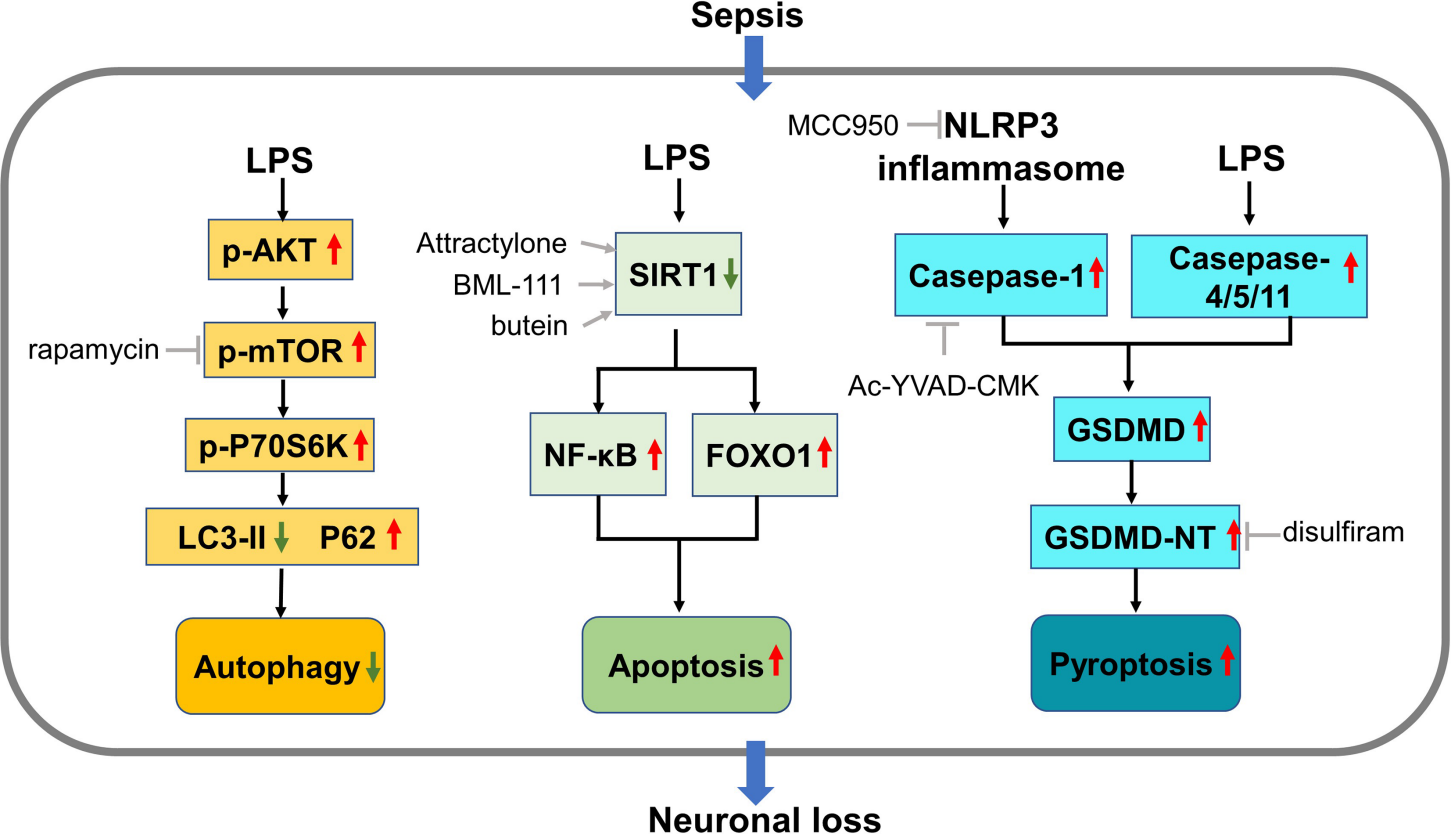
Mechanisms of neuronal loss in sepsis. During sepsis, the AKT/mTOR pathway is activated and inhibits the normal autophagy process in neurons, leading to autophagic neuronal death. Suppression of SIRT1 by LPS activates NF-κB and FOXO1, triggering neuron apoptosis. Inflammasome generated in sepsis activates casepase-1 while LPS activates casepase-4/5/11, both of which promotes the generation of GSDMD. Cleavage of GSDMD produces GSDMD-NT which forms membrane pores and lead to neuronal pyroptosis. LPS, lipopolysaccharides; mTOR, mammalian/mechanistic target of rapamycin; LC3, SIRT1, silent information regulator 1; FOXO1, forkhead box protein O1; GSDMD, gasdermin D; GSDMD-NT, GSDMD-N-terminal products.

Accumulating evidence has provided mechanistic insights into neuronal loss in sepsis and subsequent long-term cognitive impairment. Apoptosis (119), autophagy (120), and pyroptosis (121) have been identified as possible mechanisms of neuronal loss in sepsis (**Figure 2**). In response to sepsis, mitochondrial dysfunction, and over-production of ROS, apoptosis and autophagy are stimulated to regulate the death of neuronal cells (122). Indeed, by consuming and recycling macromolecules and damaged organelles, autophagy restores physiological ROS level and maintains cellular homeostasis. Alternatively, when ROS is produced excessively, apoptosis will be initiated to clear damaged cells (123).

It has been acknowledged that enhancing autophagy may prevent cognitive impairment in sepsis by reducing neuronal cell damage (124). One of the most widely investigated pathways implicated in this process is the AKT/mTOR pathway. Generally, it participates in the neuronal development, survival and functioning of mature neurons, and is implicated in neuronal death in brain injury and diseases (125). Specifically, this pathway plays an indispensable role in the initiation of autophagy (123). For example, when CLP caused neuronal loss and subsequent long-term cognitive impairment, it upregulated the phosphorylation of key components of the AKT/mTOR pathway in the hippocampal neurons of mice, which suggested the activation of this pathway and suppression of autophagy. Concomitantly, the autophagy markers such as P62 and LC3 were reduced by CLP, indicating suppression of autophagy. Intriguingly, administration of rapamycin, an inhibitor of the AKT/mTOR pathway, rescued spatial memory by restoring the autophagic activity (107). The same team later reported that, along with the long-term neuronal loss in the CA1 region, the Akt/mTOR signaling was increasingly activated over time (from 14 to 60 days) after CLP (108). However, rapamycin was only effective when it’s used at 14 days but not 60 days after CLP, indicating that the neuronal death is irreversible and timely intervention is necessary. Inconsistently, other authors pointed out that the Akt/mTOR signaling was suppressed in the CLP model (126). In addition, by restoring suppressed Akt/mTOR signaling, recombinant human erythropoietin exerted neuroprotective effect and rescued the spatial memory 1 week after CLP. Moreover, the beneficial outcomes of exogenous recombinant human erythropoietin were abolished by inhibiting this pathway by using rapamycin (126). These contradictory results suggest that researchers should deeply explore the complicated role of Akt/mTOR in autophagy in sepsis.

Apoptotic neurons are repeatedly observed in the hippocampus of CLP model, contributing to permanent neuronal loss and cognitive impairment (127–130). Moreover, pre-treatment with medications that inhibit caspase-dependent apoptosis can attenuate the cognitive dysfunction 7 days after CLP (129). Sirtuins are histone deacetylases implicated in numerous cellular processes. As a member of the sirtuin family, silent information regulator 1 (SIRT1) plays an important role in the modulation of apoptosis (131, 132). Intriguingly, several teams consistently revealed that SIRT-activating agents can alleviate long-term cognitive impairment after sepsis by reducing neuronal apoptosis (109, 130, 133).

Pyroptosis is a rapid form of programmed cell death mediated by gasdermin D (GSDMD). It can be categorized into a canonical pathway mediated by casepase-1 and a noncanonical pathway mediated by casepase-4/5/11. In the canonical pathway, inflammasomes generated in response to external stimuli promotes the maturation of casepase-1. Casepase-1 then promotes the maturation of IL-1β and IL-18. Meanwhile, Casepase-1 further cleaves GSDMD into N- and C-terminal components (GSDMD-NT and -CT). GSDMD-NT can insert into and form pores in plasma membranes, leading to lytic cell death and secretion of mature IL-1β and IL-18. Similarly, in the noncanonical pathway, cytosolic LPS is sensed by casepase-4/5/11, which are in turn activated and cleave GSDMD into GSDMD-NT. In sepsis, both pathways can be activated (134). The NOD-like receptor and pyrin domain containing 3 (NLRP3) is one of the most-explored inflammasomes involved in the canonical pathway (135). Yang et al. (136) found that prophylactic delivery of either NLRP3 inhibitor or casepase-1 inhibitor before CLP surgery protected CA1 neurons from degeneration and thus preserved learning and memory function of mice 2 weeks later. Even 3 months after LPS stimulation, the knockout of NLRP3 protected neuronal structure including dendritic arbors and spines as well as synaptic plasticity (137). A recent report evidenced that disulfiram, an alcohol deterrent, can interfere with GSDMD-mediated pore-forming, making it an attractive medication for treating sepsis-associated long-term cognitive impairment (138).

## Conclusion

**Figure 3:**
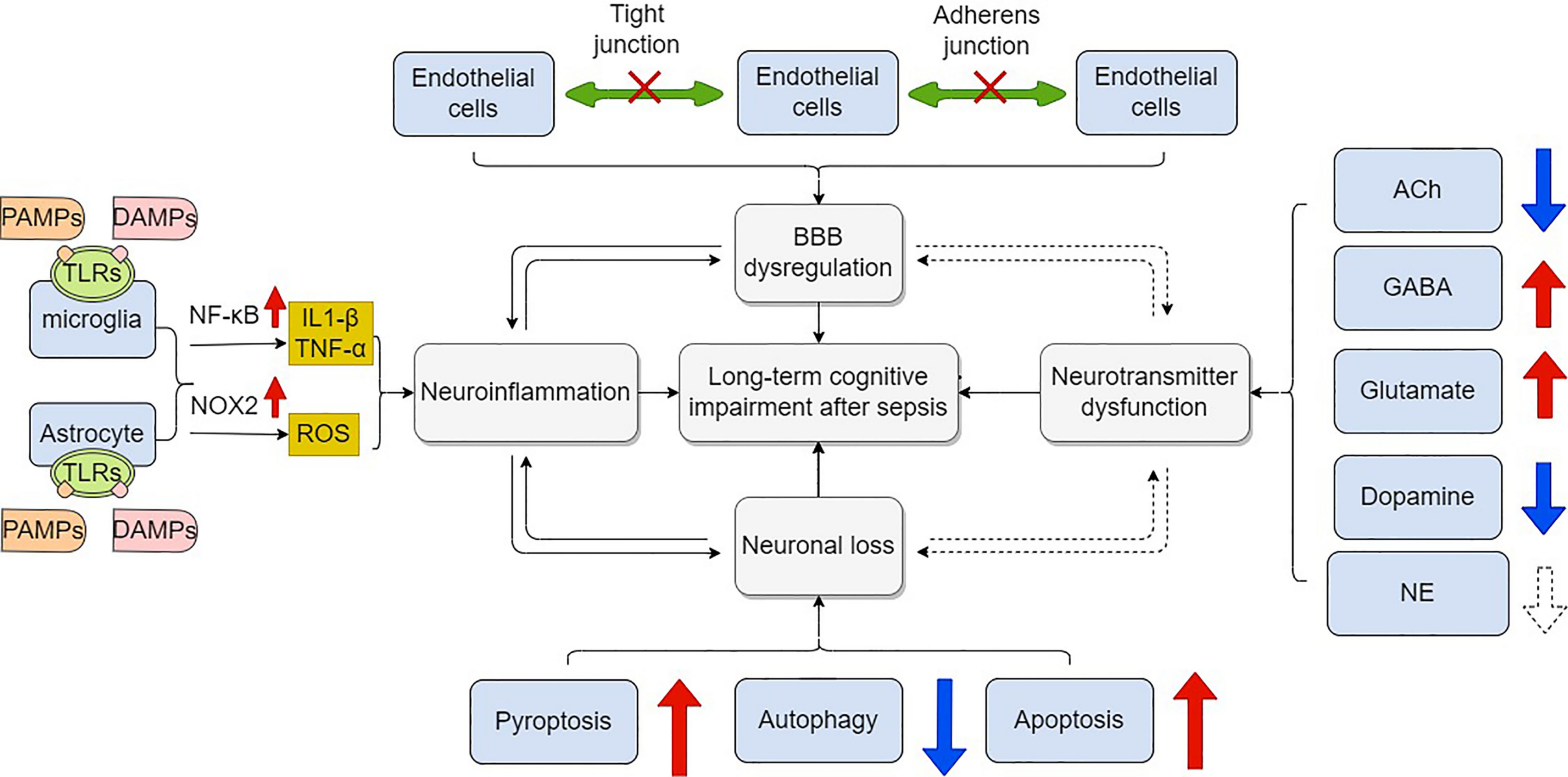
Mechanisms of long –term cognitive impairment after sepsis. Blood brain barrier is composed of endothelial cells, astrocytes and pericytes. These cells are connected by tight junctions, adherens junctions and gap junctions. Dysfunction of these components leads to increased permeability of BBB in response to sepsis, which facilitates long-term cognitive impairment. Microglia and astrocyte are over-activated mainly through the activation of NF-κB signaling when PAMPs and DAMPs including LPS by binding to their ligands (namely, TLRs) on cell membrane. Pro-inflammatory cytokines are subsequently over-produced while anti-inflammatory cytokines are down-regulated. Neuroinflammation further leads to cognitive impairment. Overproduction of ROS is medicated by NOX2 in activated microglia and astrocytes. Neuronal loss is common in sepsis. Apoptosis, autophagy, and pyroptosis are involved in the modulation of neuronal death. Finally, dysregulation of neurotransmitters including ACh, GABA, glutamate, dopamine and NE also contributes to cognitive impairment in sepsis. BBB, blood brain barrier; PAMPs, pathogen-associated molecular patterns; DAMPs, damage-associated molecular patterns; LPS, lipopolysaccharide; TLRs, toll like receptors; ROS, reactive oxidative species; NOX2, NADPH oxidase 2; ACh, acetylcholine; GABA, Gamma-aminobutyric acid; NE, norepinephrine.

Like dementia, long-term cognitive impairment after sepsis is almost irreversible, which decreases life quality of patients and increases economic burden. When treating septic patients, clinicians should be alert to this sequela and act as early as possible. As for the mechanisms, long-term cognitive impairment is profoundly associated with a cascade of several detrimental events, including BBB disruption, neuroinflammation, neurotransmitter dysfunction, and neuronal loss (**Figure 3**). Based on these mechanisms, various interventions have been tested in numerous animal studies. The positive results indicate that targeting certain critical steps of those pathologic events will be promising for preventing and treating long-term cognitive impairment after sepsis. Despite significant advances having been made in our understanding of sepsis -related long-term cognitive impairment, several questions remain. Firstly, the complex interplay among these mechanisms should be taken into consideration when designing pre-clinical and clinical studies. Secondly, there has been some criticism about the translational value of current animal models of sepsis, particularly for the CLP model (139). Finally, there is currently no consensus on the timelines for long-term cognitive assessment after sepsis. Addressing these important issues will significantly improve our understanding of long-term cognitive impairment after sepsis.

## Author Contributions

YL drafted the first version of the manuscript. MJ revised the manuscript. JY approved the final manuscript. All authors contributed to the article and approved the submitted version.

## Funding

This work was supported by the grants from the National Natural Science Foundation of China (81772126, 81971892, 81971020).

## Conflict of Interest

The authors declare that the research was conducted in the absence of any commercial or financial relationships that could be construed as a potential conflict of interest.

## Publisher’s Note

All claims expressed in this article are solely those of the authors and do not necessarily represent those of their affiliated organizations, or those of the publisher, the editors and the reviewers. Any product that may be evaluated in this article, or claim that may be made by its manufacturer, is not guaranteed or endorsed by the publisher.
